# The sequential mediation model of students’ willingness to continue online learning during the COVID-19 pandemic

**DOI:** 10.1186/s41039-022-00188-w

**Published:** 2022-03-25

**Authors:** Abdul Hafaz Ngah, Nurul Izni Kamalrulzaman, Mohamad Firdaus Halimi Mohamad, Rosyati Abdul Rashid, Nor Omaima Harun, Nur Asma Ariffin, Noor Azuan Abu Osman

**Affiliations:** 1 Faculty of Business, Economics and Social Development, Universiti Malaysia Terengganu, 21030 Kuala Nerus, Terengganu Malaysia; 2 Centre for Academic Management and Quality, Universiti Malaysia Terengganu, 21030 Kuala Nerus, Terengganu Malaysia; 3 Centre for Foundation and Continuing Education, Universiti Malaysia Terengganu, 21030 Kuala Nerus, Terengganu Malaysia; 4 Data and Digital Development Centre, Universiti Malaysia Terengganu, 21030 Kuala Nerus, Terengganu Malaysia; 5 Faculty of Science and Marine Environment, Universiti Malaysia Terengganu, 21030 Kuala Nerus, Terengganu Malaysia; 6 Faculty of Fisheries and Food Science, Universiti Malaysia Terengganu, 21030 Kuala Nerus, Terengganu Malaysia; 7 Faculty of Engineering, University of Malaya, 50603 Kuala Lumpur, Malaysia

**Keywords:** Online learning, COVID-19, Stimulus–organism–response theory, Willingness to continue

## Abstract

This study explored the factors influencing students’ willingness to continue with the online learning system during the coronavirus disease 2019 (COVID-19) pandemic by adopting the stimulus–organism–response (SOR) theory. This study also incorporated e-learning readiness, performance, and satisfaction as mediators. The present study employed the purposive sampling method, whereby 2215 data of undergraduate students from a public university were gathered using an online survey and analysed using structural equation modelling (SEM) with Smart Partial Least Squares (SmartPLS). The results revealed that students’ e-learning readiness, performance, and satisfaction positively influenced their willingness to continue online learning. Besides, students’ e-learning readiness, performance, and satisfaction sequentially mediated the relationship between the online learning system quality and willingness to continue online learning. Significantly, this study provided new insights into the literature on students’ willingness to continue online learning by providing empirical evidence on the factors that support their willingness to continue online learning during the COVID-19 pandemic.

## Introduction

The COVID-19 pandemic has shaken and impacted activities around the world. Most notably, this infectious virus has affected the education sector globally (Dhawan, 2020) across all education levels. Governments worldwide were forced to closed educational institutions to prevent the spread of COVID-19 (UNESCO, 2019). In light of this situation, social distancing is vital to contain the spread of this particular disease; hence, education sectors are struggling to adapt to the challenges caused by it. Consequently, higher education institutions worldwide were forced to shift the learning method from regular physical classrooms to online learning methods to ensure smooth teaching and learning. Malaysia also opted to use online learning as a communication method to replace face-to-face learning. Presently, online learning is the most popular alternative solution to prevent a COVID-19 outbreak on campuses (Ating, 2020).

Essentially, online educational technology provides an interactive virtual classroom environment, enabling students to engage with the efficiency of a classroom without the need for physical presence (Bawaneh, 2021). Generally, online educational technology offers organisation resources, and only certain applications offer a virtual classroom environment. Although there was significant growth and adoption in education technology before the pandemic, a notable surge of usage in virtual tutoring, video conferencing, and online learning tools were recorded since the COVID-19 pandemic (Li & Lalani, 2020). Despite Malaysia having a technology literacy rate of 95% for the age group of 15 years and older (UNESCO, 2021), it still faced challenges with the online learning approach as it is not fully implemented such as in more developed country (Ating, 2020). Hence, studies on online learning in Malaysia are vital to benefit policy makers in improving any shortcomings. Besides, transitioning from a physical classroom to an online learning method is overwhelming for students and educators, especially if the implementation of online learning system is not clear (Motte, 2013).

In normal circumstances, students enrol in the universities with a full belief that they would learn by face to face with professors in the campus. Nevertheless, COVID-19 has forced all education institutions worldwide to pursue their education online, regardless of proper mental and physical preparation. However, since students’ expectations and the real scenario are entirely different, their willingness to continue to rely on online learning is questionable. It is noteworthy to mention that there is limited literature on the willingness to continue their study online during the COVID-19 outbreak. Thus, there is a crucial need to identify the factors that influence their willingness to continue online learning. Furthermore, Almaiah and Alismaiel (2019) and Garba Shawai and Amin Almaiah (2018) stated that the success of the online learning method relies on students’ willingness and comprehensive understanding of adoption factors and challenges of the online learning system.

There are numerous studies on the importance of willingness as a factor influencing the success of the online learning system. Nevertheless, the studies involved different settings, such as life before the COVID-19 pandemic (Christensen & Knezek, 2017; Kaufmann & Tatum, 2018; Tsai et al., 2018). Hence, understanding the factors influencing students’ willingness to continue using the online learning system is vital since students still rely on this alternative solution during the outbreak. The current study focused on a single public institution, due to the different platforms used in other universities and varying user experiences.

Furthermore, this study referred to the SOR framework by Mehrabian and Russell (1974) to investigate the factors influencing students’ willingness to continue using the online learning system by operationalising system quality as the ‘stimulus’, electronic learning (e-learning) readiness, performance, and satisfaction as the ‘organism’, and willingness to continue as the ‘response’. The implication from this study is essential for higher education institutions to improve the quality of online learning system. Moreover, since most learning institutions depend on the online learning method during the pandemic, the findings of this study could serve as a reference to enhance students’ e-learning readiness and performance to ensure their willingness to continue using the online learning system.

## Literature review and framework development

### Stimulus–organism–response theory

The SOR theory was first introduced in 1974 by Mehrabian and Russell. This theory proposed that stimuli (S) from an individual’s environment could affect the cognitive and affective response (O), leading to behavioural response (R) (Mehrabian & Russell, 1974). Moreover, Baghozzi (1986) proposed that these stimuli are a person’s external elements of the physical atmosphere, whereas an organism refers to a person’s internal processes and structures occurring in between stimuli and responses. This theory is suitable in this study’s context since it has been used in researching online learning (Yang et al., 2019, 2021; Zhao et al., 2020). According to Yang et al. (2019), the SOR theory explains college students’ decision-making process in mobile learning continuance by incorporating perceived autonomy, perceived competence and perceived relatedness as stimulus. The stimulus in the study affected the organism of cognitive and affective learning involvement thus will positively influence the students’ continuance intention. In addition, the study showed that cognitive learning environment and affective learning environment explain the variance of 0.712 in mobile learning continuance intention, hence explaining 71.2% of the total variance in continuance intention. The SOR model also explains e-learning continuance among college students during the pandemic. The literature by Zhao et al. (2020) which applied flow theory in the SOR model influences massive open online course (MOOC) continuance. The study proposed that stimuli of interactivity, media richness, and sociability could affect telepresence and social presence. Subsequently, the stimulus will be affecting the organism of flow thus influencing continuance intention in MOOC setting. Observably, the study showed that the construct of flow in the SOR model explained 37% of the total variance in continuance intention, hence explaining the student’s behaviour. The amount of variance or *R*^2^ value is explained by the number of predictors in a research model (Tuan Mansor et al., 2021). Hence, differences in amount of variance between Yang et al. (2019) and Zhao et al. (2020) could be explained by the different number of predictors in respective studies.

According to Ngah, Anuar, et al. (2021), the flexibility of the SOR theory allows researchers to develop their model based on their context of studies as long as it adheres to the original stimulus–organism–response concept. Thus, based on that, the SOR theory has been modified according to the context of the study. Following the proposed idea by Mehrabian and Russell (1974), current study stipulates system quality as stimuli since it comes from students’ surrounding environment using the online learning system and influences their internal response, i.e. their e-learning readiness, performance, and satisfaction. The last stage of the model explained that organism will influence the response. Hence, students with a high level of satisfaction and e-learning readiness who also believe that their performance will increase while using the online learning system could have a higher willingness to continue using the online learning system.

### Willingness to continue

The term willingness has been explained by Gibbons and Gerrard (1995) as the acceptance of an individual to engage the behaviour under certain conditions. In contrast with intention, willingness does not require consideration anticipated consequences of a behaviour. For instances, individual who is willing to participate in dangerous behaviour responds to risk-inducing circumstances rather than creating them. Willingness to continue happens after the initial consumption of a product or service, which indicates that it is acceptable to a customer, and they are likely to continue using it. This idea can be a proxy of behavioural intentions, and thus, it has been adopted in academia (Shin et al., 2017). On the other hand, willingness to use indicates behavioural intention before the actual consumption, whereas willingness to continue is the behavioural intention after the initial use.

The literature provided that willingness to continue or continuance intention has been tested in other theoretical frameworks such as in expectation confirmation model by Dai et al. (2020), the integration of personality trait and motivational theory by Alabdullatif and Velázquez-Iturbide (2020) and in self-regulated learning perspective by Zhu et al. (2020). Hence, the investigation of students’ continuance intention appears to focus on diverse aspects. Therefore, the current study identified the factors influencing students’ willingness to continue using the online learning system. Students’ e-learning readiness, performance, and satisfaction are incorporated in a SOR model to determine their willingness to continue using the online learning system. The SOR theory suitable to delve into the external stimuli and emotional reactions explains the future individual response (Ngah, Rahimi, et al., 2021).

### Online learning system quality

System quality is defined as an individual’s perception of a system (Kurt, 2019). The literature has provided that system quality is an essential factor for a good user experience (Ahn et al., 2004). System quality in the online learning system is measured by hardware availability and various software applications designed for an intended need (Freeze et al., 2010). Besides, a successful online learning system requires internet access to enable network-to-network communication. System quality also includes characteristics such as availability, usability, dependability, performance, and functionality (Sarrab et al., 2015).

This study submitted that the online learning system quality positively affects students’ e-learning readiness, performance, and satisfaction. Kaur and Abas (2004) defined e-learning readiness as an individual’s ability to use online learning resources and multimedia technologies to improve learning quality. It should be noted that the relationship between system quality and e-learning readiness was positively confirmed by Al-araibi et al. (2019), who highlighted the importance of hardware in e-learning readiness. Based on the literature, the proposed hypothesis is as follows:

H1: Online learning system quality positively influences students’ e-learning readiness.

Perceived performance is the student’s ability to complete a task and respond to the learning environment (Chang et al., 2014). Generally, online learning system quality influences the user performance, as confirmed by Ali and Younes (2013) and Chirchir et al. (2019), who revealed the positive relationship between system quality and individual performance. Hence, this study proposed the following hypothesis:

H2: Online learning system quality positively influences students’ performance.

Satisfaction in education is the students’ perception of the learning experience and how the environment aids academic success (Lo, 2010). Studies regarding online learning have confirmed the positive relationship between system quality and satisfaction, as elaborated in Cidral et al. (2018) and Li et al. (2020). Therefore, the current study posited that:

H3: Online learning system quality positively influences students’ satisfaction.

### E-learning readiness

E-learning readiness is an individual’s ability to utilise online learning resources and multimedia technologies in order to enhance learning quality (Kaur & Abas, 2004). Meanwhile, readiness in online learning is defined by three aspects; 1) students’ preference on the form of delivery compared to face-to-face classroom instruction; 2) students’ confidence in using electronic communication for learning; and 3) the ability to engage in autonomous learning (Warner et al., 1998). According to Demir (2015), online learning readiness is a crucial indicator in completing online classes successfully.

Previous literature provided that students’ e-learning readiness to use the online learning system influences their performance and willingness to continue. The positive relationship between e-learning readiness and performance in online learning was discovered by Wei and Chou (2020). Thus, the following hypothesis is presented:

H4: Students’ e-learning readiness positively influences students’ performance.

The positive relationship between online learning readiness and willingness to continue was confirmed in (Gupta & Maurya, 2020). Hence, this study presents the following hypothesis:

H5: Students’ e-learning readiness positively influences students’ willingness to continue.

### Perceived performance

Students’ performance is their ability to complete a task and their response towards the learning environment (Chang et al., 2014). In this study, positive performance of using the online learning system develops positive willingness to continue.

Studies have shown that performance has a positive influence on the willingness to continue. According to Shahijan et al. (2018), students’ perceived performance positively influences continuance intention. Hence, the following hypothesis is posited:

H6: Students’ performance positively influences willingness to continue.

### Satisfaction

Satisfaction is the effect of being satisfied with a positive feeling, whereas dissatisfaction is a negative feeling (Bhattacherjee, 2001). In view of this, satisfaction with prior experience influences an individual’s intention to continue using a system or service (Bhattacherjee, 2001). In education, satisfaction is the students’ perception of the learning experience and how the learning environment facilitates their academic success (Lo, 2010). In the context of this study, students’ satisfaction with prior experience influences their performance and willingness to continue.

Furthermore, the relationship between satisfaction and perceived performance has been confirmed by Otto et al. (2020) and Philip and Moon (2013). Therefore, the proposed hypothesis is as follows:

H7: Students’ satisfaction positively influences students’ performance.

Essentially, studies have confirmed the positive relationship between satisfaction and continuance intention. In the academic setting, the relationship between satisfaction and continuance intention of online learning was verified by Joo et al. (2018) on the Korean Massive Open Online Course, Cheng and Yuen (2020) on secondary students’ continuance, and Cheng (2020) on cloud-based e-learning systems. Hence, the study proposed following hypothesis:

H8: Students’ satisfaction positively influences students’ willingness to continue.

### Mediation

Mediation analysis plays a crucial role in model enhancements and theoretical advancement (Ngah et al., 2020). The SOR concept explains that the variable representing the organism could also be a mediator in the study (Lockwood & Pyun, 2019). Therefore, this study aimed to enhance the model’s predictive power by adding students’ e-learning readiness as a mediator for the relationship between online learning system quality and willingness to continue and between online learning system quality and performance. Additionally, performance is added as a mediator between the relationship of e-learning readiness and willingness to continue.

As emphasised above, Al-araibi et al. (2019) have confirmed the positive relationship between system quality and e-learning readiness while Chirchir et al. (2019) confirmed the relationship between e-learning readiness and performance. Moreover, students’ e-learning readiness and performance also showed a positive relationship with willingness to continue (Gupta & Maurya, 2020; Shahijan et al., 2018). Furthermore, Veeramootoo et al. (2018) and Masri et al. (2020) mentioned that system quality has a positive relationship with user continuance. Referring to the model, it is highly likely that the mediators (e-learning readiness and performance) are in a causal relationship with continuance intention. Hence, the following hypothesis is proposed:

H9: Students’ e-learning readiness and performance mediate the relationship between system quality and students’ willingness to continue.

Moreover, studies have corroborated the relationship between system quality and satisfaction (Li et al., 2020) and the relationship between performance and willingness to continue (Shahijan et al., 2018). On another note, the positive relationship between system quality and continuance intention was confirmed by Masri et al. (2020). Considering that satisfaction and performance are in a causal relationship between system quality and willingness to continue, the current study postulated the following hypothesis:

H10: Students’ satisfaction and performance mediate the relationship between system quality and students’ willingness to continue.

**Fig. 1:**
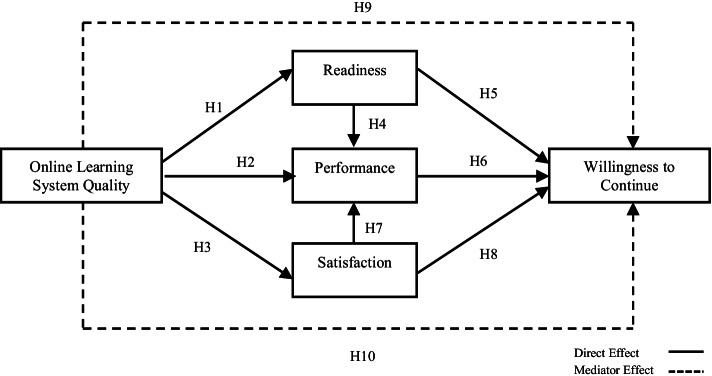
Research framework

Figure 1 illustrates the research framework of the study.

## Research methodology

### Instrument development

In this study, the questionnaire was divided into two parts: 1) the demographic profile of the respondents and 2) the measurement items of five latent constructs mentioned in the research model. All the construct items were adopted from previous studies and modified accordingly. The measurement items for the online learning system quality and satisfaction were adopted from Isaac et al. (2019). Moreover, items of e-learning readiness were adopted from Mirabolghasemi et al. (2019), while items of performance were adopted from Damnjanovic et al. (2013). Finally, items measuring willingness to continue were based on Bhattacherjee (2001). Podsakoff et al. (2003) proposed using a different anchor scale to measure independent and dependent variables as a procedural method to remedy the CMV. Besides, a similar scale would increase the perceived similarity and redundancy of items to encourage respondents to be less thorough in item comprehension, memory retrieval, and judgement. These detrimental tendencies can be reduced by varying the scale types and anchor labels (MacKenzie & Podsakoff, 2012). Hence, a five-point Likert scale was used following (Ngah, Gabarre, et al., 2021) to measure the independent variables, whereas a seven-point Likert scale was used to measure the dependent variables to reduce the CMV.

### Sampling and data collection

This study applied the purposive sampling method since it focused on degree students attending courses through online learning systems during the second semester of 2019/2020. The degree students represent the major population of the public institution and thus representing the actual situation. The survey used Google Forms, distributed online via the Official Facebook for one month at the end of the semester. According to Ngah, Ramayah, et al. (2019), Ngah, Thurasamy, et al. (2019)), the sample size can be determined using the power of analysis based on the number of predictors. Gefen et al. (2011) suggested that with a power of 80%, medium effect size, and *p* = 0.05, the minimum sample size of the study should be 91. Ultimately, 2,215 completed questionnaires were returned; hence, sample size was not an issue in this study.

The findings indicated that most respondents were female (79.3%), while (20.7%) were male. Furthermore, a majority of the respondents were second-year students, 34.3% were first-year students, 25.3% were third-year students, and 2.6% were fourth-year students. Most respondents were from the Faculty of Business, Economics and Social Development (32.1%), 20.9% were from the Faculty of Science and Marine Environment, 19.9% were from the Faculty of Ocean Engineering Technology and Informatics, 19.1% were from the Faculty of Fisheries and Food Science, and 7.9% were from the Faculty of Maritime Studies. Besides, the respondents were between 21 and 25 years old. As for the bachelor’s degree programme, the entry requirements are pre-university and diploma qualification. In terms of the electronic device usage of the respondents, 78.8% used more than one electronic device, 15.1% use laptops, 5.3% use smartphones, and only 0.8% use desktops. Additionally, the findings revealed that most of the respondents (83.2%) have internet access at home, whereas 16.8% do not have internet access (Table 1).

**Table 1:** Respondent profile

Variable	Item	Frequency (N = 2215)	Percentage (%)
Gender	Male	459	20.7
	Female	1756	79.3
Year of study	First year	759	34.3
	Second year	837	37.8
	Third year	561	25.3
	Fourth year	58	2.6
Faculty	Faculty of Maritime Studies	175	7.9
	Faculty of Science and Marine Environment	464	20.9
	Faculty of Ocean Engineering Technology and Informatics	440	19.9
	Faculty of Fisheries and Food Science	424	19.1
	Faculty of Business, Economics and Social Development	712	32.1
Electronic device	Laptop	334	15.1
	Desktop/PC	17	0.8
	Smartphone	119	5.3
	More than 1 device	1745	78.8
Internet access at home	Yes	1843	83.2
	No	372	16.8

### Data analysis

This study aimed to predict the relationship between variables in the research model; hence, the data were analysed using Smart PLS (Ringle et al., 2015), a co-variance-based SEM. The present study also referred to Hair et al. (2019) and applied the two-stage approach: 1) the measurement model, which assesses the convergent validity and discriminant validity, and 2) testing the structural model using the bootstrapping method with 5,000 resampling technique (Hair et al., 2019), implemented in testing the hypotheses.

### Common method variance

This study used single-source data in which the dependent variable and the independent variables were answered by the same person simultaneously; hence, procedural and statistical methods were employed to overcome issues related to CMV (MacKenzie & Podsakoff, 2012; Ngah, Ramayah, et al., 2019; Ngah, Thurasamy, et al., 2019). The procedural methods that were applied have been discussed in the section on instrument development. For the statistical method, based on Podsakoff et al. (2012) the unmeasured marker variable technique was employed to test the CMV.

Meanwhile, the unmeasured marker variables were used as an exogenous variable predicting every endogenous variable in the model. All the significant effects of the model without the marker variable remained significant in the model with the marker variable. Notably, no significant common method bias was found in the data, indicating that CMV is not an issue in this study.

### Measurement model

For the measurement model, the criteria for convergent validity and discriminant validity must be fulfilled. Generally, convergent validity can be established if the loading reaches a value of 0.708 or higher (Hair et al., 2019), average variance extracted (AVE) reaches a value of 0.5, and composite reliability (CR) achieves a minimum value of 0.7 (Hair et al., 2019). Table 2 shows that the convergent validity is acceptable because AVE and CR are higher than the threshold values, thus confirming that the convergent validity is not an issue in the study.

**Table 2:** Convergent validity

Variable	Loading	CR	AVE
Online learning system quality	0.905	0.942	0.844
	0.927		
	0.924		
E-learning readiness	0.919	0.945	0.812
	0.891		
	0.875		
	0.919		
Performance	0.950	0.950	0.865
	0.962		
	0.875		
Satisfaction	0.950	0.968	0.909
	0.954		
	0.956		
Willingness to continue	0.961	0.973	0.923
	0.963		
	0.959		

After the convergent validity was fulfilled, the discriminant validity of the model was tested. Discriminant validity is confirmed if the heterotrait–monotrait (HTMT) values are lower than 0.9 (Franke & Sarstedt, 2019). The results depicted in Table 3 satisfy the HTMT criterion, indicating that all the values were lower than the proposed 0.9. Hence, the results in this study proved that the model met the discriminant validity requirements of the tested constructs and items.

**Table 3:**
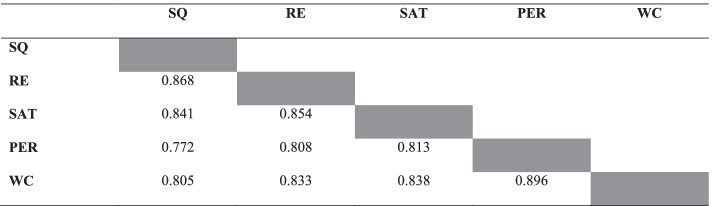
HTMT

*SQ* online learning system quality; *RE* e-learning readiness; *SAT* satisfaction, *PER* performance; *WC* willingness to continue

### Structural model

**Fig. 2:**
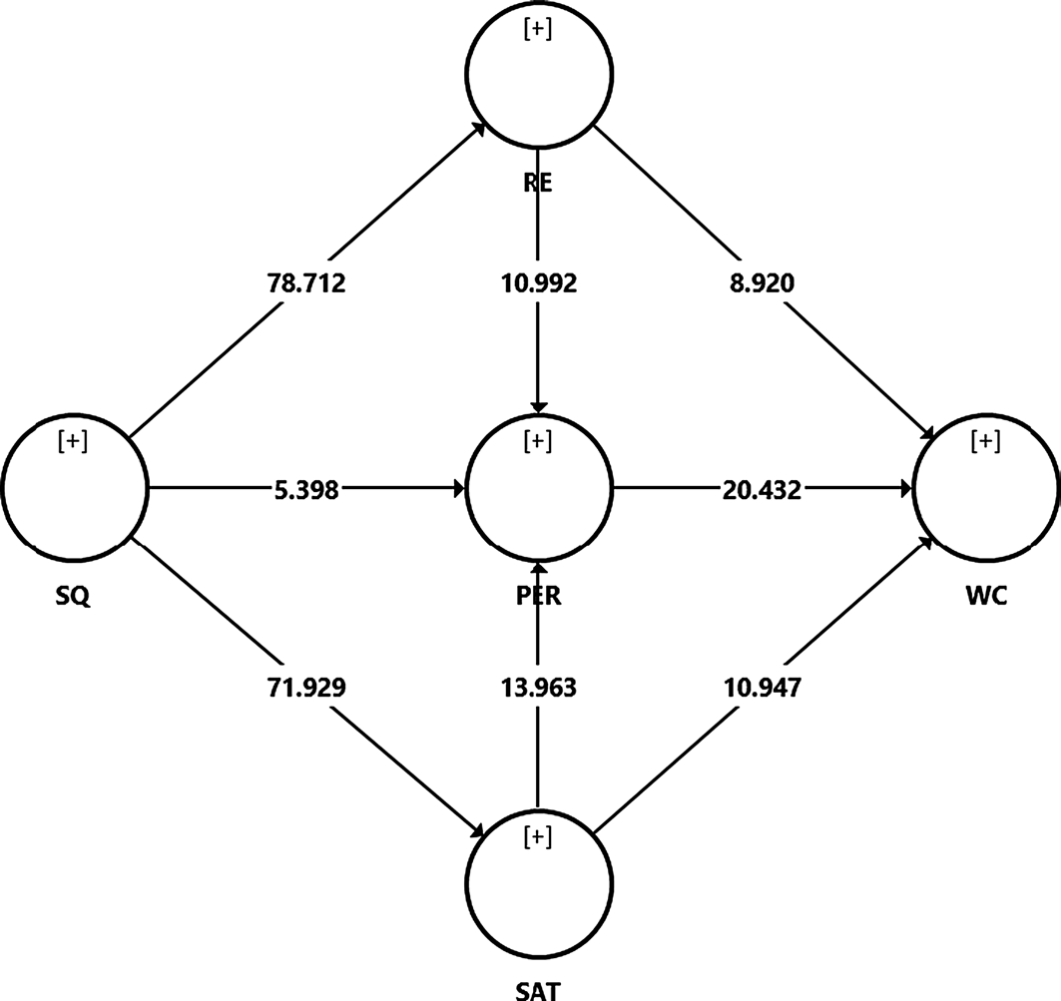
Structural model

After the measurement model assessment, the multicollinearity test was performed to ensure no collinearity issues in the model before evaluating the structural model. Collinearity was assessed with the variance inflated factor (VIF) values, which must be lower than the threshold value of 5 (Hair et al., 2017). Table 4 shows that all the VIF values were less than five, indicating no collinearity problem between the predictor variables. Subsequently, hypothesis testing was conducted by applying a bootstrapping technique. Figure 2 demonstrates the structural model of the study.

**Table 4:** Hypotheses testing

Hypothesis	Relationship	Beta	SE	*T* value	*p* value	LL	UL	VIF
H1	SQ—> RE	0.796	0.010	78.712	0.000	0.779	0.812	1.000
H2	SQ—> PER	0.149	0.028	5.398	0.000	0.102	0.192	3.228
H3	SQ—> SAT	0.781	0.011	71.929	0.000	0.762	0.798	1.000
H4	RE—> PER	0.312	0.028	10.992	0.000	0.265	0.359	3.540
H5	RE—> WC	0.221	0.025	8.920	0.000	0.182	0.264	3.207
H6	PER—> WC	0.482	0.024	20.432	0.000	0.442	0.520	2.718
H7	SAT—> PER	0.395	0.028	13.963	0.000	0.348	0.442	3.321
H8	SAT—> WC	0.256	0.023	10.947	0.000	0.216	0.293	3.368

*SQ* online learning system quality; *RE* e-learning readiness; *SAT* satisfaction; *PER* performance; *WC* willingness to continue

Table 4 provides the results for hypothesis testing, whereby all the direct effect hypotheses were supported. As for H1 whereby it was posited that online learning system quality (SQ) has a positive influence on e-learning readiness (RE), the results showed a positive relationship (*β* = 0.796, *t* = 78.712: lower level (LL) = 0.779, upper level (UL) = 0.812, *p* < 0.001). Therefore, H1 was supported. For H2, it was suggested that SQ positively influences perceived performance (PER), and the results showed that SQ had a positive relationship on PER (*β* = 0.149, *t* = 5.398: LL = 0.102, UL = 0.192, *p* < 0.001). Therefore, H2 was supported. Regarding H3, it was proposed that SQ positively influences satisfaction (SAT), and the results showed that SQ had a positive relationship on SAT (*β* = 0.781, *t* = 71.929: LL = 0.762, UL = 0.798, *p* < 0.001). Therefore, H3 was supported.

For H4 and H5 on the relationship between RE, PER, and willingness to continue (WC) (*β* = 0.312, *t* = 10.992: LL = 0.265, UL = 0.359, *p* < 0.001) and (*β* = 0.221, *t* = 8.920: LL = 0.182, UL 0.264, *p* < 0.001), respectively, the values confirmed positive relationships between RE, PER, and WC, hence supporting H4 and H5. Meanwhile, H6 showed that PER had a positive influence on WC (*β* = 0.482, *t* = 20.432: LL = 0.442, UL 0.520, *p* < 0.001). Thus, H6 was supported. Finally, the relationship between SAT, PER, and WC was supported with (*β* = 0.395, *t* = 13.963: LL = 0.348, UL = 0.442, *p* < 0.001) and (*β* = 0.256, *t* = 10.947: LL = 0.216, UL 0.293, *p* < 0.001). Consequently, H7 and H8 were supported.

Table 5 presents the values of predictive relevance (*Q*^2^) through the blindfolding technique and effect size (*f*^2^). The *R*^2^ values of 0.639 for PER, 0.634 for RE, 0.610 for SAT, and 0.785 for WC indicate that RE, SAT, and SQ explained 63.9% of the variance for PER. On the other hand, SQ explained 63.4% of the RE variance, whereas SAT explained 61.0% of the SAT variance. Lastly, PER, RE, and SAT explained 78.5% of the WC variance. As for predictive relevance, a value of *Q*^2^ higher than 0 indicates that the model has good predictive relevance (Hair et al., 2017).

**Table 5:** Predictive relevance (*Q*^2^) and effect size (*f*^2^)

Relationship	*Q*^2^	*f*^2^	Decision
RE—> PER	0.549	0.076	Small
SAT—> PER		0.130	Small
SQ—> PER		0.019	Small
SQ—> RE	0.510	1.729	Large
SQ—> SAT	0.551	1.561	Large
PER—> WC	0.720	0.398	Large
RE—> WC		0.071	Small
SAT—> WC		0.090	Small

*SQ* online learning system quality; *RE* e-learning readiness; *SAT* satisfaction; *PER* performance; *WC* willingness to continue

From the blindfolding technique, the study found that *Q*^2^ is 0.549, 0.510, 0.551, and 0.720 for PER, RE, SAT, and WC, confirming that the model has good predictive power for the subject matter in this study. Lastly, the effect size of *f*^2^ was assessed, in which an effect size of 0.02 is considered small, 0.15 is moderate, and a value above 0.35 is considered high (Cohen, 1988). It can be observed that RE, SAT, and SQ have a small effect size towards PER with a value of 0.076, 0.130, and 0.019. With a value of 1.729 and 1.561, SQ has a large effect size on RE and SAT. For WC, the study found that PER (0.398) has a large effect on WC. In contrast, RE (0.071) and SAT (0.090) have a small effect on WC.

For the mediation analysis, Preacher and Hayes (2008) and Hair et al. (2017) suggested bootstrapping the indirect effect to test the mediation effect. Preacher and Hayes (2008) also stated that LL and UL do not straddle a 0 in between and indicated a mediation effect is non-existent between independent and dependent variables. The results in this study presented that (*β* = 0.120, *t* = 8.855: LL = 0.094, UL 0.147, *p* < 0.001) for the relationship between SQ—> RE—> PER—> WC, confirming that RE and PER has a sequential mediation effect on the relationship between SQ and WC, hence supporting H9. As for H10, the results indicated that SAT and PER sequentially mediated the relationship between SQ and WC (*β* = 0.149, *t* = 12.503: LL = 0.126, UL 0.174, *p* < 0.001). Table 6 shows the results of the mediation effect in this study.

**Table 6:** Mediation analysis

Hypothesis	Relationship	Beta	SE	*T* value	*p* value	LL	UL
H9	SQ—> RE—> PER—> WC	0.120	0.014	8.855	0.000	0.094	0.147
H10	SQ—> SAT—> PER—> WC	0.149	0.012	12.503	0.000	0.126	0.174

*SQ* online learning system quality; *RE* e-learning readiness; *SAT* satisfaction; *PER* performance; *WC* willingness to continue

## Discussion

The present study has proven that online learning system quality positively affects students’ e-learning readiness (H1). These findings are aligned with Al-araibi et al. (2019), who revealed that hardware availability positively influences students’ e-learning readiness. Hence, the results indicated that a good quality online learning system affects student’s e-learning readiness to use the online learning system.

This study also revealed that students’ online learning system quality positively impacts students’ performance (H2), in line with Ali and Younes (2013) and Chinchir et al. (2019). It was also discovered that online learning system quality (H3) influences students’ satisfaction. This finding signalled the importance of online learning system quality in improving students’ e-learning readiness, performance, and satisfaction. Therefore, the university’s management should create an effective and user-friendly online learning system to ensure students are ready to study in a new norm using the online learning system. Most importantly, students’ e-learning readiness with the online learning system could increase their performance and satisfaction. A good quality online system should be updated with the current requirements and technology to ensure educators could share and deliver the content effectively. On top of that, good technical support should be provided to ensure the system is applicable for both students and educators. Without proper support, the full potential of the online system cannot be utilised by users.

As for H4 and H5, students’ e-learning readiness influenced students’ performance and willingness to continue online learning, consistent with the findings in Wei and Chou (2020) and Gupta and Maurya (2020). For H6, students’ performance had positively impacted students’ willingness to continue online learning. The results in this study aligned with Shahijan et al. (2018), who highlighted that performance positively affects students’ willingness to continue online learning. Lastly, for the direct effect relationship, students’ satisfaction influenced students’ performance and willingness to continue online learning (H7 and H8). The study corroborated with Otto et al. (2020), Philip and Moon (2013), who found that students’ satisfaction positively affects students’ performance. This result also supported the findings in Cheng and Yuen (2020) and Cheng (2020). Therefore, UMT’s management should be proactive in creating a positive attitude among students to complete their online classes and improve performance by providing a good online learning system during the COVID-19 pandemic. Hence, the lecturers and university management must ensure their students are prepared to face the new norm of students’ lives during the pandemic and are willing to continue using online learning.

In terms of the mediation effect (H9), the current study confirmed that students’ e-learning readiness and performance mediate the relationship between online learning system quality and students’ willingness to continue online learning. Lastly, the mediation analysis showed that satisfaction and performance sequentially mediate the relationship between online learning system quality and the students’ willingness to continue online learning (H10). Thus, this finding proved that online learning system quality is crucial in determining students’ intention to continue online learning. In conclusion, the UMT management and lecturers should ensure the compatibility of the online learning system to increase students’ e-learning readiness, satisfaction, and performance. Most importantly, e-learning readiness, satisfaction, and the students’ performance improvement while using the online learning system positively affect their continued usage of online learning.

## Conclusion

The importance of the online learning system during the COVID-19 outbreak should not be underestimated. Most universities rely on the online learning system to ensure the students could continue their learning process. Thus, this study proposed a framework based on the SOR theory to determine the contributing factors of students’ willingness to continue online learning in UMT with e-learning readiness, satisfaction, and performance as sequential mediators. In light of the current COVID-19 situation in Malaysia, this study provided new insights on the literature concerning students’ willingness to continue online learning by providing empirical evidence on the factors that support the willingness to continue online learning among university students.

### Theoretical implications

The current study provides a new SOR model to explain the effect of the factors on students’ willingness to continue using online learning in the COVID-19 pandemic setting. By introducing new combination factors of the SOR model, this study enriched the SOR model to explain students’ willingness to continue using the online learning system. Using online learning system quality as a stimulus mediated by students’ e-learning readiness and performance is one of the earliest proposed strategies in online learning, particularly during the COVID-19 pandemic. Due to this outbreak, online learning is practised as the new norm in Malaysian higher education institutions; therefore, it is critical to understand the factors that could influence students’ positive behaviour towards online learning. The study found that online learning system quality can predict e-learning readiness, performance and satisfaction, and the willingness to continue using online learning systems in a single framework. Additionally, the study confirmed that e-learning readiness, performance, and satisfaction mediate the relationship between online learning system quality and willingness to continue.

### Practical implications

In terms of practical implication, the findings help the university’s management enhance the online learning system to improve students’ e-learning readiness and willingness to continue using online learning. Besides, the implementation of online learning during the COVID-19 pandemic will be a milestone in exposing university students to Education 4.0. Hence, lecturers and university management play a vital role in preparing students to handle the new teaching and learning process. The findings indicated that online learning system quality is a vital factor influencing the students’ willingness to continue. Therefore, the university's information technology (IT) department should address problems quickly during the online class to ensure the students learn without any distractions. Besides, as the learning method focuses on online learning, the university should improve the support service in the IT department by providing better training for the technicians. Technicians well versed in an online learning environment could significantly improve the support services. Thus, maintaining an excellent online learning system quality is crucial as it influences students’ e-learning readiness, performance and satisfaction, and willingness to continue.

### Limitations and future research

Even though the present study has provided new insight into knowledge, the limitations should also be considered. This study only focused on UMT students; thus, future studies should include students from other higher education institutions in Malaysia to investigate further students’ willingness to continue online learning. The study discovered that the students are willing to continue with the online learning method using the SOR theory. Hence, future studies should extend the framework by incorporating new influencing factors to understand students’ willingness to continue using online learning system. Besides, future studies should use other theories to determine students’ willingness to continue. The study proposed that Duckworth et al.’s (2007) ‘grit’ should be included in future studies to better understand students’ behaviour in online learning environment. In addition, future studies should also identify factors that could hinder students from using online learning during the COVID-19 pandemic.

## Data Availability

The data cannot be shared; please contact the corresponding author for getting the access to the dataset.

## Abbreviations

AVE: Average variance extracted
COVID-19: Coronavirus disease 2019
CR: Composite reliability
HTMT: Heterotrait–monotrait
IT: Information technology
LL: Lower level
PER: Perceived performance
RE: E-learning readiness
SAT: Satisfaction
SE: Standard error
SEM: Structural equation modelling
SOR: Stimulus–organism–response
SQ: Online learning system quality
UL: Upper level
VIF: Variance inflation factor
WC: Willingness to continue
